# Standardizing Patient-Derived Organoid Generation Workflow to Avoid Microbial Contamination From Colorectal Cancer Tissues

**DOI:** 10.3389/fonc.2021.781833

**Published:** 2022-01-10

**Authors:** Mattia Marinucci, Caner Ercan, Stephanie Taha-Mehlitz, Lana Fourie, Federica Panebianco, Gaia Bianco, John Gallon, Sebastian Staubli, Savas D. Soysal, Andreas Zettl, Stephan Rauthe, Jürg Vosbeck, Raoul A. Droeser, Martin Bolli, Ralph Peterli, Markus von Flüe, Charlotte K. Y. Ng, Otto Kollmar, Mairene Coto-Llerena, Salvatore Piscuoglio

**Affiliations:** ^1^ Visceral Surgery and Precision Medicine Research Laboratory, Department of Biomedicine, University of Basel, Basel, Switzerland; ^2^ Institute of Medical Genetics and Pathology, University Hospital Basel, Basel, Switzerland; ^3^ Clarunis, Department of Visceral Surgery, University Centre for Gastrointestinal and Liver Diseases, St. Clara Hospital and University Hospital Basel, Basel, Switzerland; ^4^ Institute of Pathology, Viollier AG, Allschwil, Switzerland; ^5^ Department for BioMedical Research, University of Bern, Bern, Switzerland

**Keywords:** patient-derived organoids, colorectal cancer, Primocin, antibiotics, microbial contamination control

## Abstract

The use of patient-derived organoids (PDO) as a valuable alternative to *in vivo* models significantly increased over the last years in cancer research. The ability of PDOs to genetically resemble tumor heterogeneity makes them a powerful tool for personalized drug screening. Despite the extensive optimization of protocols for the generation of PDOs from colorectal tissue, there is still a lack of standardization of tissue handling prior to processing, leading to microbial contamination of the organoid culture. Here, using a cohort of 16 patients diagnosed with colorectal carcinoma (CRC), we aimed to test the efficacy of phosphate-buffered saline (PBS), penicillin/streptomycin (P/S), and Primocin, alone or in combination, in preventing organoid cultures contamination when used in washing steps prior to tissue processing. Each CRC tissue was divided into 5 tissue pieces, and treated with each different washing solution, or none. After the washing steps, all samples were processed for organoid generation following the same standard protocol. We detected contamination in 62.5% of the non-washed samples, while the use of PBS or P/S-containing PBS reduced the contamination rate to 50% and 25%, respectively. Notably, none of the organoid cultures washed with PBS/Primocin-containing solution were contaminated. Interestingly, addition of P/S to the washing solution reduced the percentage of living cells compared to Primocin. Taken together, our results demonstrate that, prior to tissue processing, adding Primocin to the tissue washing solution is able to eliminate the risk of microbial contamination in PDO cultures, and that the use of P/S negatively impacts organoids growth. We believe that our easy-to-apply protocol might help increase the success rate of organoid generation from CRC patients.

## Introduction

Colorectal cancer (CRC) is the fourth most common and the third most deadly cancer worldwide (1). Studies exploring the genetic and epigenetic landscape of CRC (2, 3) have revealed a high level of clonal heterogeneity (4), impairing treatment opportunities. Therefore, the use of an accurate and reliable model system that allows the analysis of genotype-to-phenotype correlations for these patients is needed. One major development in the last 10 years is the development of patient-derived organoids (PDO) (5). The establishment of organoid technology has remodeled the *in vitro* culture platform for biomedical research, thus creating a powerful resource for pre-clinical studies (6). Indeed, PDOs have been shown to recapitulate the cellular heterogeneity of the primary tissue and can be maintained long term while retaining genetic stability (7). Moreover, several studies have validated the accuracy and sensitivity of PDOs to predict treatment response in CRC (7–10). In this scenario, generating a PDO biobank may create an unprecedented opportunity to fill in the existing gap between cancer genetics and patient trials, complement cell lines and xenograft-based drug studies and allow personalized therapy design (11, 12).

Based on the initial protocol for production of self-renewing intestinal organoids by Sato et al. (13), a more specific protocol for the generation of organoids from human colon and colorectal adenoma tissue was developed and reported (14, 15). Generation of CRC-PDOs can be divided into two main steps: (i) tissue dissociation using mechanical or enzymatic digestion, and (ii) culture of Matrigel-embedded tissue-derived cell suspension in medium containing CRC specific growth factors. The success rate of CRC organoid generation ranges from 55% to 90% (11, 16–18). The definition of the optimal culture conditions, as well as of the appropriate matrix, have been key factors in increasing the success rate (11, 19). However, tissue processing and culture conditions still need optimization to further increase the success rate of CRC-PDO generation.

PDO generation has been shown to be impaired by bacterial contamination (11), especially when no antibiotics are used during the washing steps prior to tissue dissociation (11, 18). Colon and rectum are microbiota-containing organs (20), therefore with an implicit risk of microbiota contamination for the PDO culture. Addition of antibiotics and antimycotics to the culture medium is the most common way to prevent microbial contamination (11). In the context of CRC-PDOs, there are no common guidelines, and the protocol regarding the addition of antibiotics differs among laboratories (9, 11, 18, 21–23), with Primocin (InvivoGen, #ant-pm-1), penicillin/streptomycin (P/S), or their combination being the most commonly described (9, 11, 18, 21–23). In particular, a study by Otte et al. used a CRC-PDO growth medium containing antibiotics/antimycotics, which was switched after 48 h with a medium containing only 100 U/ml penicillin and 100 µg/ml streptomycin (22). On the other hand, Yao et al. (9) implemented PDOs culture medium with Normocin (Invivogen, #ant-nr-1) and gentamicin/amphotericin B (GIBCO, #R01510), while Miyoshi et al. (24) employed P/S together with plasmocin (Invivogen, #ant-mpp). This variety of media compositions used in the generation of CRC PDOs highlights the need for a unified approach in order to generate robust, reproducible findings.

While efforts have been made to reduce the risk of bacterial contamination through the addition of antibiotics to culture medium, optimization of additional washing steps prior to tissue processing may also help to reduce this risk. The utility of washing steps prior to tissue dissociation has been reported; however, there is a lack of standardization regarding protocol and reagents (10, 11, 25, 26). The study of Ooft et al. (10) described the collection of CRC tissue in medium supplemented with P/S without washing steps, leading to bacterial contamination in 5% of the PDO. Similarly, another study (11) showed that including washing steps with PBS reduced the microbial contamination to 15% of the cases. A recent study from Costales-Carrera et al. (27) reported that, besides a limited amount of biopsy material, bacterial contamination limited the success rate of PDOs derivation to 74%.

In this study, we sought to prevent the risk of contamination in CRC-PDOs by proposing an optimized and easy-to-apply washing protocol prior to tissue dissociation. Here, we showed that washing surgically resected CRC tissues with Primocin-containing PBS eliminates the risk of microbial contamination. Additionally, we found that the use of P/S as a washing solution negatively impacts the success rate for CRC-PDO generation. We believe that use of our standardized, simple protocol could help increase the success rate of organoid generation from CRC patients.

## Materials and Methods

### Patient Cohort

Ten human colon and six rectal tissues were obtained from 16 patients undergoing surgery at the University Center for Gastrointestinal and Liver Disease (Clarunis), Basel, Switzerland. Written informed consent was obtained from all patients. The study was performed in accordance with the Helsinki Declaration and approved by the ethics committee (Ethics Committee of Basel, EKBB, no. 2019-02118). Data were collected retrospectively in a non-stratified and non-matched manner including patient age, sex, treatment, American Joint Committee on Cancer (AJCC) clinical stage, bowel preparation, and antibiotic prophylaxis (**Table 1**).

**Table 1:** Clinical information of the patients included in the study.

Patient ID	Tissue	Age	Sex	Treatment	AJCC Clinical	Bowel preparation	Antibiotic prophylaxis	Tumor size (cm)	Ulcer	Stenosis grade
101	Colon	64	M	None	IIIb	Yes	Cefazolin 2 g + Metronidazole 500 mg	7.5 × 6 × 2	No	2
115	Colon	64	M	None	IIIb	No	Cefazolin 2 g + Metronidazole 500 mg	4, 8	Ulcer	Non-stenotic
116	Colon	73	F	None	IIIb	Yes	Cefazolin 2 g + Metronidazole 500 mg	2.2 × 2 × 1.3	No	Non-stenotic
118	Colon	87	M	None	IVa	No	Cefuroxim 3 g	6.5 × 5 × 3.5	Ulcer	Stenotic; whole circumferential growth
119	Colon	34	F	None	IIIc	No	Cefuroxim 3 g + Metronidazole 500 mg	2, 5	No	Stenotic
131	Colon	46	M	None	IVa	Yes	Cefazolin 2 g + Metronidazole 500 mg	6	No	Obstructive growth
221	Colon	80	F	None	IIa	Yes	Cefazolin 2 g + Metronidazole 500 mg	8 × 5.5	Central ulcer	Stenotic
224	Colon	84	M	None	IIIb	Yes	Cefazolin 2 g + Metronidazole 500 mg	3.9 × 3.5 × 1.9	No	Stenotic, no passage with instrument
225	Colon	84	F	None	IIa	Yes	Cefazolin 2 g + Metronidazole 500 mg	3.5	No	2/3 Stenotic circumferential
227	Colon	74	F	None	I	Yes	Cefazolin 2 g + Metronidazole 500 mg	3 × 2.2 × 2	No	Stenotic
204	Rectum	62	M	50.4 Gy; Capecitabine	IV	Yes	Cefazolin 2 g + Metronidazole 500 mg	4.2 × 3.5 × 1.6	Ulcer	Narrow
209	Rectum	86	M	None	IIIa	Yes	Cefazolin 2 g + Metronidazole 500 mg	2.2 × 2 × 0.5	Ulcer	1/8 of circumferential
103	Rectum	67	F	43.2 Gy; Capecitabine + Regorafenib	IIIb	Yes	Cefazolin 2 g + Metronidazole 500 mg	1 × 1 × 0.2	Yes (tumor heavy shrink)	1
107	Rectum	79	M	None	IIIb	Yes	Cefazolin 2 g + Metronidazole 500 mg	0.4 × 0.2 × 0.2	No	1/4 of circumferential
130	Rectum	81	F	None	IIIb	Yes	Cefazolin 2 g + Metronidazole 500 mg	3.5 × 3 × 0.5	No	Stenotic, no passage with instrument
135	Rectum	50	M	50.4 Gy; Capecitabine	I	Yes	Cefazolin 2 g + Metronidazole 500 mg	0.3 × 0.3 × 0.4	Ulcer	Half of circumferential

Prior to surgery, patients received a bowel preparation that combines oral antibiotics and mechanical cleansing of the colon (using a macrogol solution for cleansing) as follows: 1 day before surgery patients underwent bowel preparation and received oral antibiotic treatment of 2 500 mg tablets of Neomycin three times a day and 2 500 mg tablets of Metronidazole three times a day (16:00–20:00–24:00). Moreover, 30’ before surgery, intravenous antibiotics were administered. Patient-specific treatments are summarized in **Table 1**.

### Washing Protocol Prior Organoid Generation

**Figure 1:**
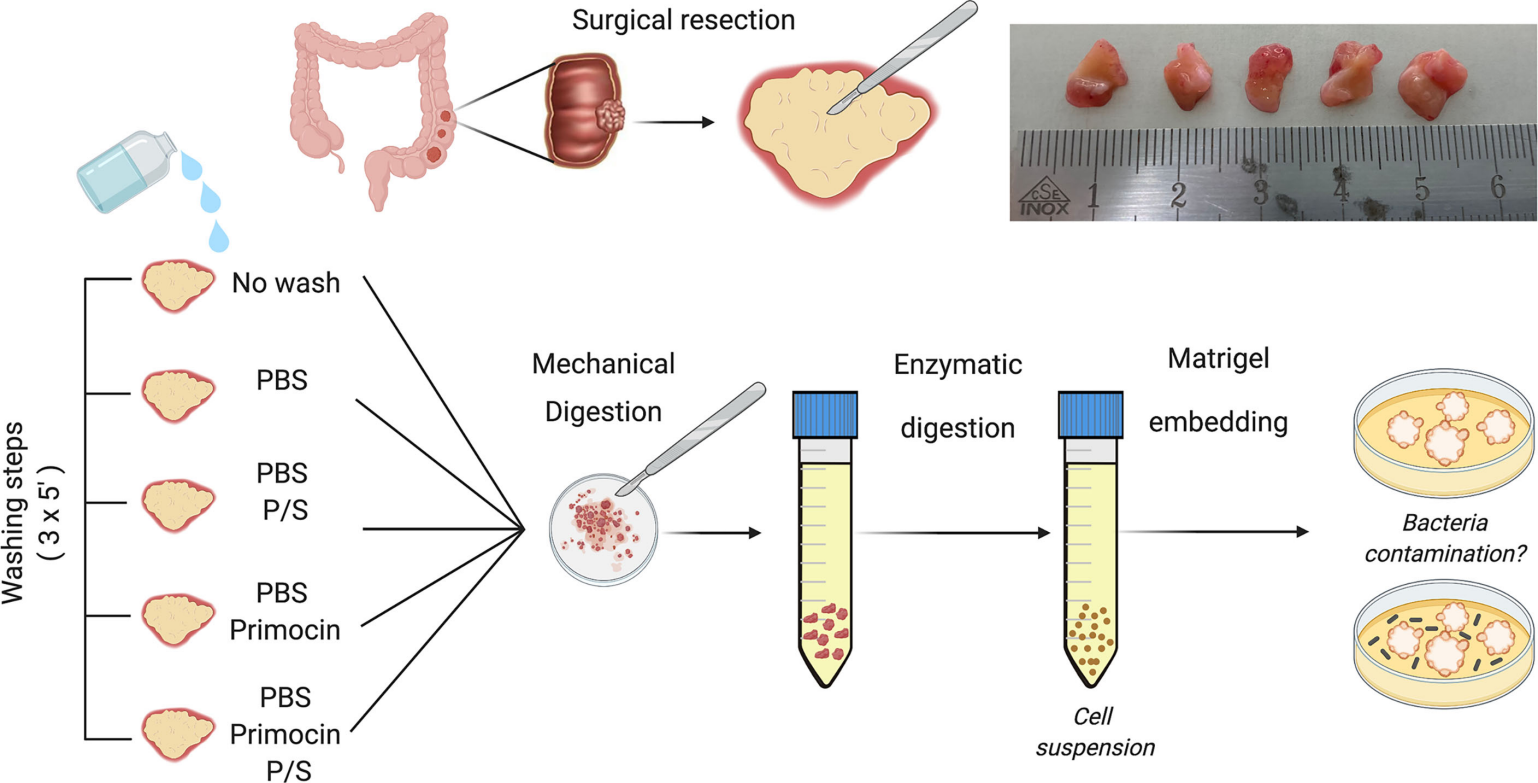
Schematic representation of the tissue processing workflow for CRC organoid generation. Upon collection, CRC tissues were divided into 5 similar pieces. Each sample underwent a different washing condition as described in the methods (left). Samples were washed three times for 5 min while maintained on ice. After the washing step, all the tissues were processed using the same protocol and conditions. A cell suspension was generated using mechanical and enzymatic digestion that was then embedded in Matrigel and daily monitored for presence of microbial contamination. Created with BioRender.com.

**Figure 2:**
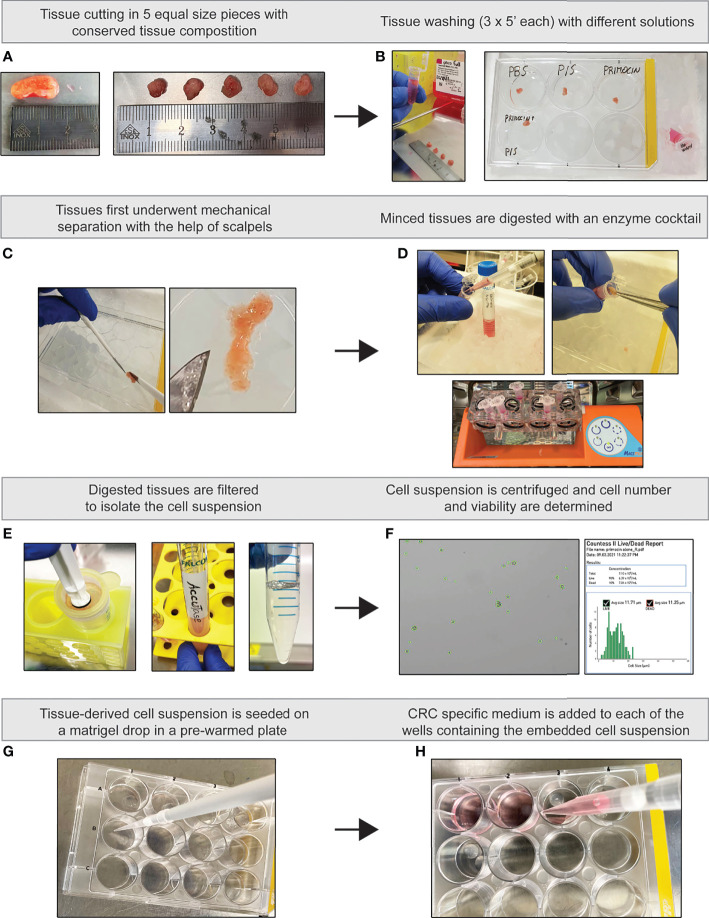
Illustrated flow chart of PDO generation. **(A)** Tissue cutting. **(B)** Washing steps. **(C)** Tissue mincing. **(D)** Enzymatic tissue dissociation. **(E)** Generation of single cell suspension. **(F)** Cell count and viability assessment. **(G)** Cell embedding in Matrigel. **(H)** Addition of supplemented PDO-CRC medium.

Human tissue samples were collected in DMEM (without any antibiotics), placed on ice, and processed after a maximum of 3 h post-surgery. The tissue was then divided into 5 pieces (**Figures 1** and **2A**), attempting to retain the tissue homogeneity regarding macroscopic tissue morphology and size among them. Subsequently, while the “no wash” condition was transferred to a 2-ml Eppendorf and kept on ice in full advanced medium for the entire washing procedure, the remaining 4 pieces were moved into 4 different wells of a 6-well plate where they were each then washed with the 4 different solutions (**Figure 2B**).

(1) No wash; the piece of tissue was kept on ice in a 2-ml Eppendorf containing Advanced DMEM/F-12 (GIBCO, #12634028) supplemented with 20 U/ml and 20 μg/ml of P/S (ThermoFisher Scientific #15140122), respectively, and Primocin (InvivoGen, #ant-pm-1) 0.1 mg/ml, Hepes (GIBCO, #15630056) 10 mM, and Glutamax (GIBCO, #35050061) 2 mM (Advanced DMEM/F-12 FULL);
(2) PBS (GIBCO, #10010023);
(3) PBS-P/S 20 U/ml and 20 μg/ml, respectively (GIBCO, #10378016);
(4) PBS-Primocin 0.1 mg/ml; and
(5) PBS-Primocin 0.1 mg/ml and P/S 20 U/ml and 20 μg/ml.

All the solutions were stored at 4°C.

Each washing solution was carefully added into the well with a serological pipette to avoid any possibility of spill-over between the different conditions until the tissues were fully covered. After 5 min, solutions were removed with a glass Pasteur pipette linked to a vacuum pump (also in this case, different pipettes were used for the 4 different conditions) and the washing was performed for 2 additional times (**Figure 2B**).

### Generation of Colorectal Cancer Patient-Derived Organoids

Following the washing steps, all tissue pieces were processed for the generation of PDOs according to standard protocol (11). Our success rate for PDO generation was 62.5%. Briefly, tissues were minced into small pieces and subsequently enzymatically digested in 1 ml of advanced DMEM/F-12 FULL medium containing 2.5 mg/ml collagenase IV (Worthington, #LS004189), 0.1 mg/ml DNase IV (Sigma, #D5025), 20 μg/ml hyaluronidase V (Sigma, #H6254), 1% BSA (Sigma, #A3059), and 10 μM LY27632 (Abmole Bioscience, #M1817) for 1 h at 37°C under slow rotation and vigorous pipetting every 15 min (**Figures 2C, D**). Of note, the DMEM used to prepare the enzymatic digestion cocktail for the “no washed” tissue was the same as where the tissue itself was kept in during the washing step. The tissue lysate was filtered through a 100 μM cell strainer, centrifuged at 300 g for 10 min, and then treated with Accutase (Sigma, #A6964) for 15 min at room temperature in order to dissociate the remaining fragments (**Figure 2E**). After 5 min of centrifugation at 300 g, the cell pellet was suspended in PBS and cells were counted using trypan blue (GIBCO, #15250061), Countess™ Cell Counting Chamber Slides (Invitrogen, #C10228) in Countess™ II FL Automated Cell Counter (Invitrogen, #AMQAF1000) (**Figure 2F**). Then, the same amount of cells from each washing condition was mixed with growth factor reduced Matrigel (Corning, #356231) and seeded as drops in a tissue-culture dish (**Figure 2G**). After polymerization of the Matrigel, a medium supplemented with growth factors for CRC tissue was added to the cells (14) (**Figure 2H**). Organoids were monitored every day for a period of 7 to 9 days for the presence of microbial contaminants. Organoid medium was changed every 3 days and, when needed, organoids were passaged after dissociation with 0.25% Trypsin-EDTA (GIBCO, #25200056).

### Immunohistochemistry Staining

Immunohistochemical staining was performed on a Benchmark immunohistochemistry staining system VENTANA BenchMark Special Stains system (Roche) using anti-CDX2 (Clone EPR2764Y, catalog number 760-4380), anti-CK20 (Clone SP3, catalog number 790-4431), and primary antibodies as substrate. Images were acquired using an Olympus BX46 microscope and evaluated by an experienced pathologist (CE).

### GRAM Staining

Bacteria detection in contaminated medium was conducted using the Remel™ Gram Stain Kit (Thermo Fisher, #R40080) following the manufacturer’s instructions. Briefly, a smear of bacteria-containing medium was placed on a glass slide and air-dried for 5 min. The smear was then quickly fixed with the flame of a Bunsen burner and stained with a crystal violet solution for 1 min at room temperature. Subsequently, the slide was rinsed under running tap water to remove excess crystal violet. Gram iodine mordant was applied for 1 min and briefly washed in tap water. To remove non-specific crystal violet staining, a Gram decolorizer solvent was applied for 30 s and then quickly rinsed under running tap water until the water ran clear. Finally, the slides were stained with Gram Safranin for 30 s and allowed to dry and then coverslipped. Images were acquired using an Olympus BX46 microscope and evaluated by an experienced pathologist (CE).

### PAS Staining

After harvesting fungi from the culture, they were briefly washed with PBS and then fixed in 10% formaldehyde overnight. Fungi was embedded in paraffin; 5-µm slices were cut and put on a slide. The staining was performed by incubating the deparaffinized slides with 0.5% periodic acid solution for 5 min, then stained with Schiff’s reagent for 10 min, followed by counterstaining with hematoxylin solution for 2 min. All steps were performed at room temperature, and slides were rinsed with tap water after each step. Images were acquired using an Olympus BX46 microscope and evaluated by an experienced pathologist (CE).

### Grocott’s Methenamine Silver Stain

As for PAS staining, we produced 5-µm slices of embedded fungi and placed them on glass slides. The staining was performed using the automatized “VENTANA BenchMark Special Stains system” from Roche and the GMS II Staining Kit (Roche, #860-028). Images were acquired using an Olympus BX46 microscope and evaluated by an experienced pathologist (CE).

### Mycoplasma Test

To check mycoplasma contamination, we performed a slightly modified PCR protocol using specific primers as described previously (28). Briefly, contaminated and uncontaminated culture medium was harvested and boiled at 95°C for 5 min or kept at 4°C up to 1 week before processing. PCR reaction was conducted with AmpliTaq Gold™ 360 Master Mix (Thermo Fisher #4398881) as previously described (28). Electrophoretic run was performed on 1.5% agarose gel and bands height were compared with TrackIt™ 100 bp DNA Ladder (Invitrogen, #10488058).

### Statistical Analysis

Statistical analyses were performed using Wilcoxon tests (Prism GraphPad 8), Spearman correlation, and Chi-square test. All statistical tests were 2-sided and *p* < 0.05 was considered statistically significant.

## Results

### Primocin-Containing Solution Prevents Organoid Culture Contamination

PBS solution, P/S, or combination of complex antibiotics have been used in washing solutions prior to tissue processing for CRC PDOs (10, 11, 25, 26). However, lack of standardization makes it difficult to conclude which is the best protocol in preventing microbial contamination. To determine the optimal components of the washing solution for CRC-PDO generation, we used a cohort of 16 CRC patients and compared the antimicrobial efficacy of 4 different solutions compared to the no-washing step: PBS, PBS supplemented with P/S, PBS supplemented with Primocin, or PBS supplemented with P/S and Primocin (**Figures 1** and **2**). The samples included in the study were mostly from male patients (56%) with a diagnosis of colon carcinoma (62.5%), and a median age of 73.5 years (range: 34–86 years). All patients included in the study received antibiotic prophylaxis on the day of the surgery (**Table 1**).

**Figure 3:**
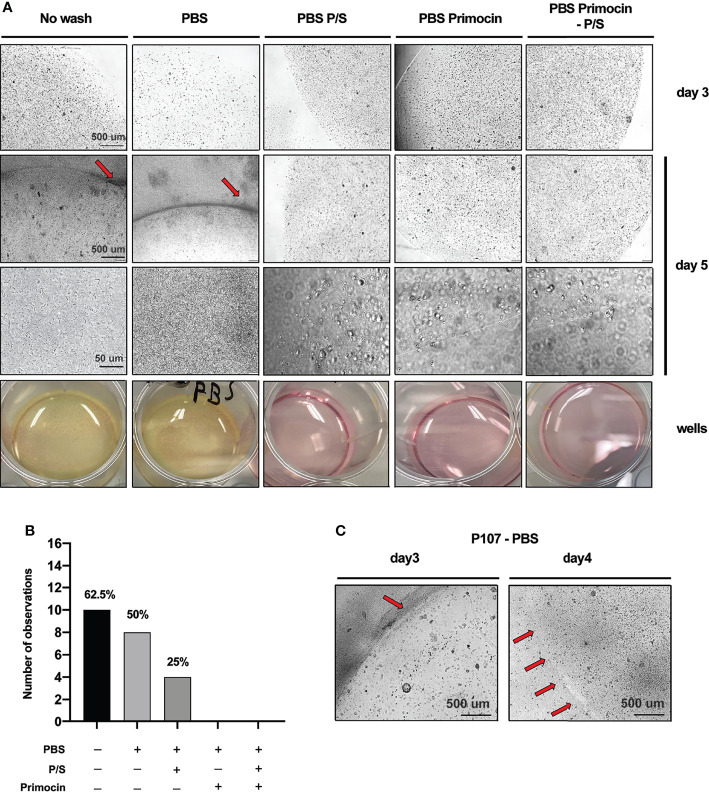
Primocin-containing washing solutions protect from bacteria contamination during organoids derivation from colorectal cancer patients. **(A)** Representative micrograph of P115 PDO cultures acquired at the microscope at day 3 and day 5. The first 2 micrographs acquired at day 5 refer to the contaminated PDO cultures and the darker area indicated by the red arrow indicates areas with high concentration of bacteria. The yellow color of the medium indicates high metabolic activity in the well due to bacteria proliferation. At high magnification, it is possible to appreciate the shape of chain-forming bacilli. **(B)** Total events observed for each washing condition. The numbers on top of the bars indicate the percentage of contaminated samples while the bars represent the absolute number of observed contaminations for each condition (within a total of 16). **(C)** Matrigel drop degradation due to bacterial contamination in P107 PDO culture. Red arrows at day 4 indicate the residue of Matrigel drop borders left after degradation, while the higher black background indicates a diffusion of bacteria.

Tissue samples were divided into 5 pieces and randomly assigned to a washing condition (**Figures 1** and **2**). All pieces underwent three washing steps on ice, for a total of 15 min, with the corresponding washing solution, with the exception of the no-wash condition (control) that was kept on ice without changing the solution. The following steps for the PDO generation, including tissue dissociation, cell seeding, and medium composition, did not differ among the washing conditions. The growth of PDOs was monitored every day and inspected for the presence of microbial contamination using an inverted microscope. Microbial contamination was usually detected between day 3 and 5 post tissue processing. In some cases (3 out of the 10 cases contaminated in the no-wash condition), the detection of microbial contamination matched a change in the color of the culture medium, indicating a change in the pH of the medium due to bacterial outgrowth. Representative images of the changes in the color of the medium and detection of bacterial contamination are shown in **Figure 3A**.

The presence of microbial contamination was detected in 62.5% of the samples that were not washed prior to tissue processing, while no contamination was detected in samples washed with solution containing Primocin (**Figure 3B** and **Table 2**). Interestingly, while washing steps performed with PBS-P/S solution reduced the risk of contamination by 37.5%, compared to the PBS alone, in 25% of tissues, microbial contamination was detected (**Figure 3B** and **Table 2**). CRC-PDO medium contains both P/S and Primocin at the same concentration used in the washing solution; thus, supplementing the culture medium is not sufficient to prevent bacteria contamination. Of note, clinical parameters such as size of the tumor or presence of ulcer were not associated with the occurrence of contamination (*p* = 0.1149, *p* = 0.6990, and *p* = 0.1432, respectively).

**Table 2:** Summary of contamination events detected for each PDO culture for each washing condition.

Contamination					Patient ID	Organoid presence				
No wash	PBS	P/S	Primocin	Primocin + P/S		No wash	PBS	P/S	Primocin	Primocin + P/S
** **	** **	** **	** **	** **	**101**	** **	** **	** **	** **	** **
** **	** **	** **	** **	** **	**115**	** **	** **	** **	** **	** **
** **	** **	** **	** **	** **	**116**	** **	** **	** **	** **	** **
	** **	** **	** **	** **	**118**	** **	** **	** **	** **	** **
** **	** **	** **	** **	** **	**119**	** **	** **	** **	** **	** **
** **	** **	** **	** **	** **	**131**	** **	** **	** **	** **	** **
** **	** **	** **	** **	** **	**103**	** **	** **	** **	** **	** **
** **	** **	** **	** **	** **	**107**	** **	** **	** **	** **	** **
** **	** **	** **	** **	** **	**130**	** **	** **	** **	** **	** **
** **	** **	** **	** **	** **	**135**	** **	** **	** **	** **	** **
** **	** **	** **	** **	** **	**204**	** **	** **	** **	** **	** **
** **	** **	** **	** **	** **	**209**	** **	** **	** **	** **	** **
** **	** **	** **	** **	** **	**221**	** **	** **	** **	** **	** **
** **	** **	** **	** **	** **	**224**	** **	** **	** **	** **	** **
** **	** **	** **	** **	** **	**225**	** **	** **	** **	** **	** **
** **	** **	** **	** **	** **	**227**	** **	** **	** **	** **	** **
**62.5%**	**50%**	**25%**	**0%**	**0%**	**Total**	**75%**	**62.5%**	**62.5%**	**87.5%**	**94%**

⬛ Positive event ⬛ Negative event

Despite the contamination, we observed healthy organoids also in the presence of bacteria. However, the bacterial contamination may be responsible for matrix drop degradation (loss of extracellular matrix support for the CRC-PDO growth), thus indicating that preventing microbial contamination is a critical step of tissue processing and organoid generation (**Figure 3C**). A comparison of Matrigel-embedded PDO derived from P107 at day 3 and day 4 after using PBS as a washing solution: at day 3, Matrigel drop borders are still intact despite bacterial contamination (dark area on the bottom right, **Figure 3C**). Inversely, at day 4, bacterial overgrowth led to Matrigel degradation, suggested by the curved border of residue from the degraded drop, indicated by the 4 red arrows. As a consequence of the degradation, while PDOs at day 3 are not surrounded by bacterial suspension, at day 4, bacteria cover all areas of the well (**Figure 3C**).

Taken together, our results indicate that implementation of multiple washing steps (3×, 5 min each) with PBS-Primocin solution prior to tissue dissociation is able to eliminate the inherent high risk of microbial contamination in CRC-PDO cultures. Moreover, we show that the presence of P/S and Primocin in the PDO culture medium is not sufficient to prevent bacterial contamination in CRC-PDO cultures.

### The Use of Penicillin/Streptomycin-Containing Solution During Tissue Washing Steps Negatively Impacts Organoid Generation

**Figure 4:**
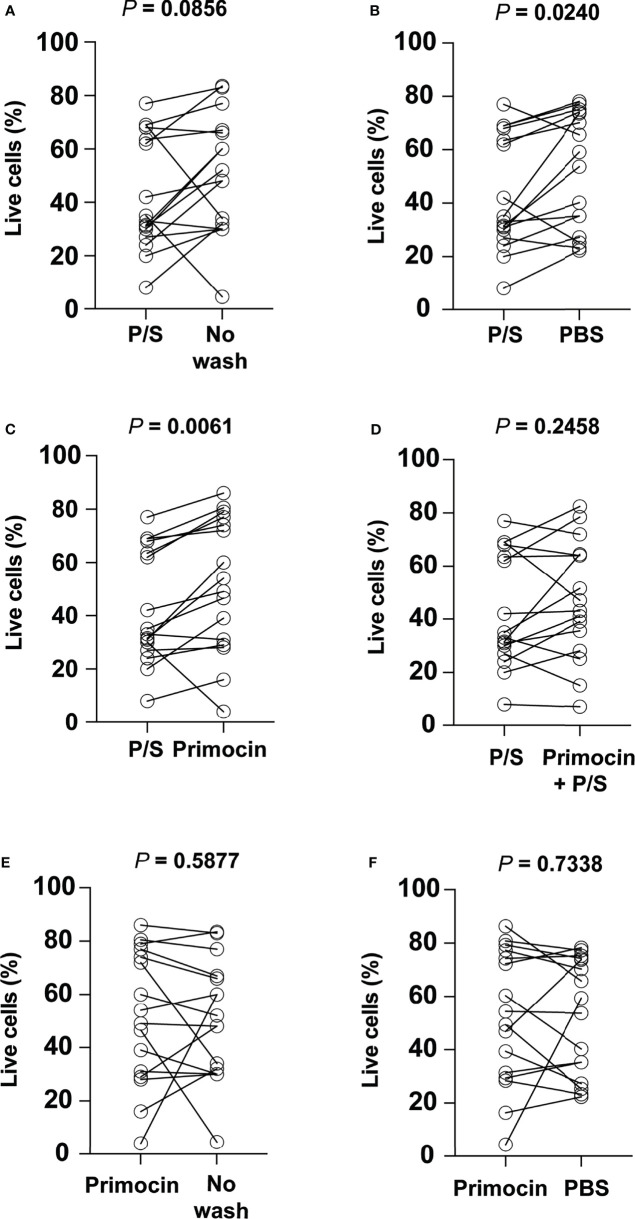
The use of penicillin/streptomycin during the washing process negatively impacts cell viability. Comparison of the percentage of viable cells obtained using penicillin/streptomycin as a washing solution with **(A)** No wash, **(B)** PBS, **(C)** Primocin, and **(D)** penicillin/streptomycin and Primocin. Further cell viability comparison between Primocin and no wash and Primocin and PBS washing conditions is shown in **(E)** and **(F)**, respectively.

To determine the impact of the additional washing steps on PDO generation, we checked cell viability immediately before embedding in Matrigel using the Trypan blue staining assay. The presence of P/S alone in the washing solution leads to a significantly lower percentage of viable cells compared to a wash performed with PBS alone and Primocin-PBS (**Figures 4A, B**; Wilcoxon test, *p* = 0.0240 and 0.0061, respectively). Likewise, washing steps performed with P/S-PBS showed a high tendency to negatively impact cell viability compared to the no-washing condition (**Figure 4C**; Wilcoxon test, *p* = 0.0856). No significant difference in cell viability was observed when comparing PBS-P/S solution and PBS-P/S-Primocin (**Figure 4D**; Wilcoxon test, *p* = 0.2458), suggesting that the presence of Primocin is not sufficient to overcome the negative impact of P/S. Moreover, no difference was noticed when comparing cell viability assessed after using other washing solutions that didn't contain P/S (**Figures 4E, F**).

Our results suggested that the presence of P/S in the washing solution could impact cell viability and therefore the success rate of CRC-PDO generation.

### Inappropriate Washing Leads to Bacterial and Fungal Contamination

**Figure 5:**
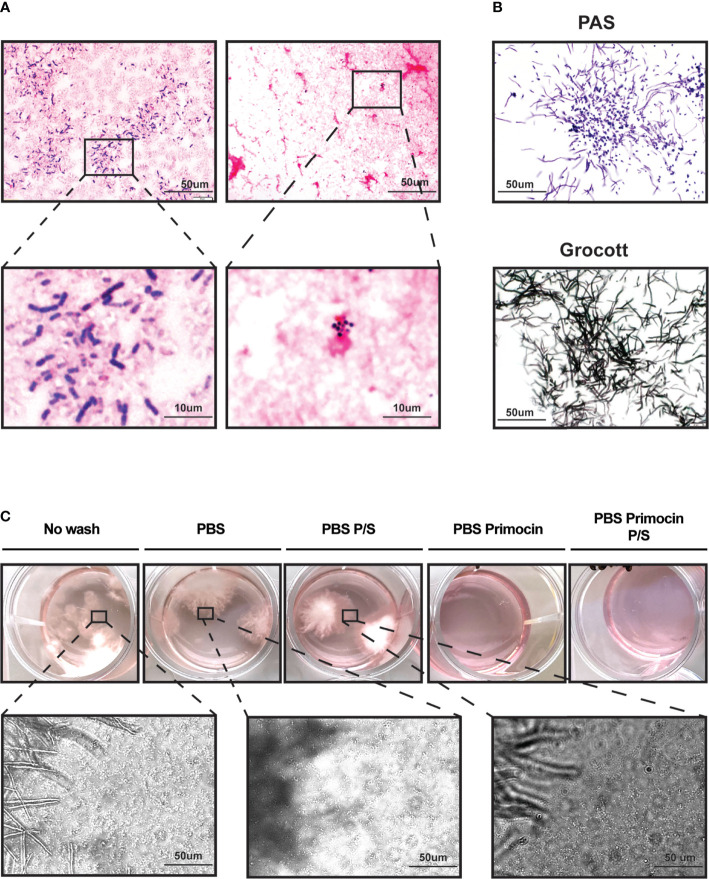
Inappropriate washing conditions lead to GRAM-positive bacteria and *Candida albicans* contamination of PDO culture. **(A)** Representative micrographs showing PDO cultures contaminated with GRAM-positive bacteria. **(B)** Yeast, hyphae, and pseudohyphae structures are attributable to *Candida albicans*. **(C)** Micrographs show simultaneous contamination of both fungi and bacteria.

To characterize the type of microbial contamination, we performed GRAM staining in the presence of a suspected bacterial contamination, and a PAS and Grocott staining when contamination appeared related to fungi. GRAM staining was consistent with the presence of GRAM-positive chain-forming bacillus and GRAM-positive cocci (**Figure 5A**). These bacteria seem to be capable of aerobic metabolism (29, 30), since they were kept in a classic incubator for cell culture where the O_2_ percentage is maintained at 18.6% (31).

Although mycoplasma is not normally present within the intestinal flora, it may be present in neoplastic disease and, if present, can alter organoid formation by affecting their structure, number, and size (32, 33). Therefore, we investigated the presence of mycoplasma contamination in the organoids generated from each washing condition. None of the organoids were positive for mycoplasma contamination (**Figure S1**).

Bacteria contamination was found to be the major contaminant in our patient-derived organoid cultures. Indeed, bacterial and fungal contamination was detected in only 1 (P135) out of the 10 cases with microbial contamination. To perform a morphological characterization of the fungi, we performed PAS and Grocott staining. We observed yeast, hyphae, and pseudohyphae structures consistent with *Candida albicans* (**Figure 5B**). Interestingly, this contamination appeared only when tissues were not previously washed with Primocin-containing PBS (**Figure 5C**), suggesting that, as for bacteria contamination, growth medium supplemented antibiotics with is not sufficient to protect cultures from fungal-yeast contamination.

### Histologic and Immunophenotypic Characterization of Generated PDO Lines

**Figure 6:**
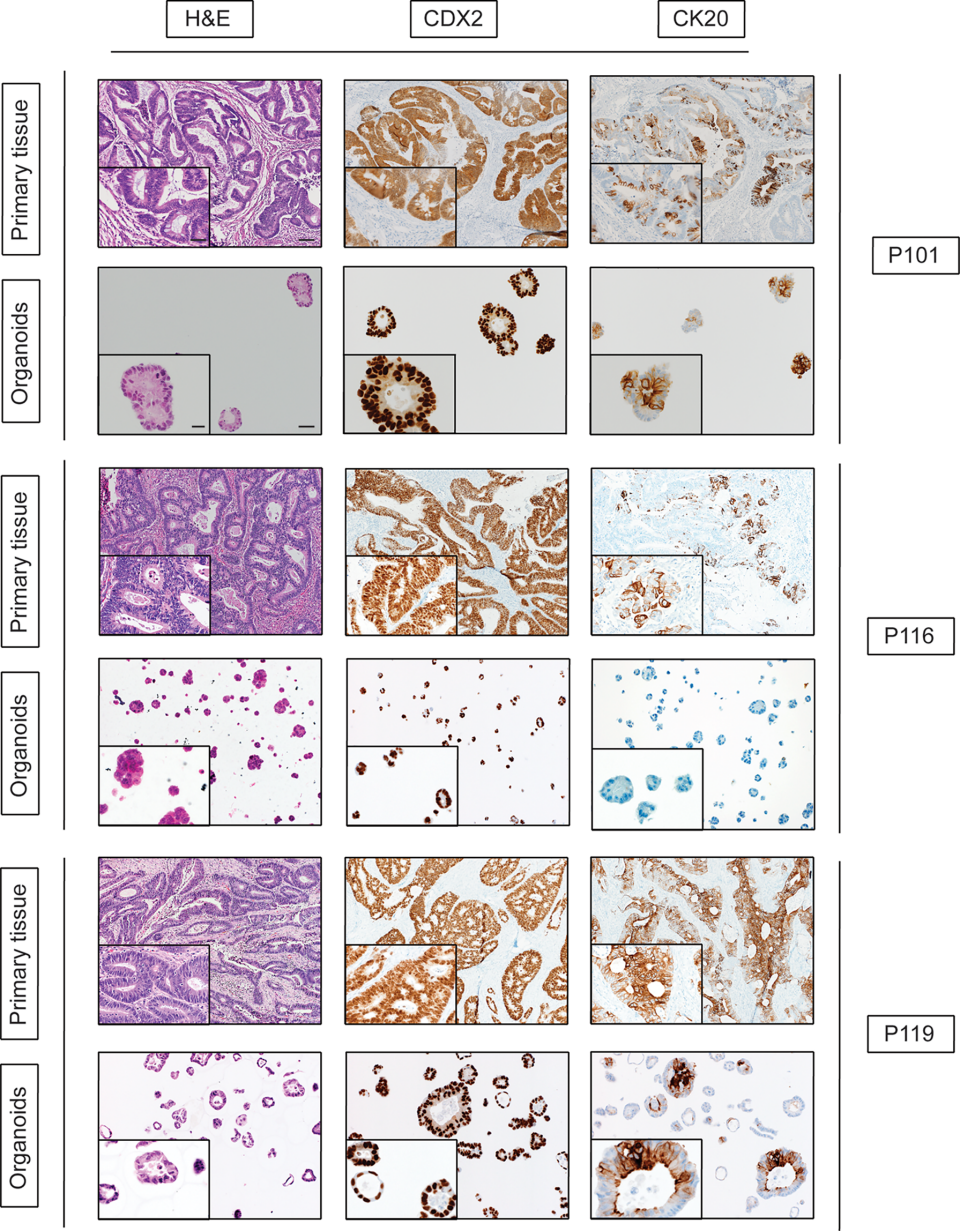
Histologic and immunophenotypic characterization of CRC organoids. Representative micrographs of matched tissue–organoids pairs. Both tissues and organoids sections were stained with hematoxylin–eosin (H&E, left side), CDX2, and CK20 antibodies. For primary tissue pictures, scale bars indicate a size of 100 µm for the picture with lower magnification and 25 µm for the one with higher magnification. For PDOs picture, scale bars are 50 µm and 10 µm for lower and higher magnification, respectively.

In our study, we generate 10 PDOs from different patients. In order to evaluate whether PDOs recapitulate primary tumor morphology and marker expression features, we performed H&E and immunohistochemistry staining. Histologic and immunophenotypic analysis was performed by an expert pathologist with expertise in gastrointestinal pathology confirming that our 10 PDOs recapitulated and maintained the histologic profile and cellular morphology of the tumors from which they originated (**Figure 6**).

## Discussion

Organoid models hold great potential for modeling human disease and providing a highly reliable tool to investigate new therapeutic approaches. Despite improvements in defining the optimal growth conditions for PDOs (34, 35), there is still space for optimization regarding tissue processing that might help to increase the success rate of CRC PDO generation.

In normal conditions, the human colon is covered by a mucus layer that consists of an inner gel-like layer, and a loose outer layer. The outer mucus layer serves as a semipermeable network providing a habitat for commensal microorganisms, while the inner gel-like mucus layer acts as a physical barrier excluding microorganisms from direct contact with the epithelium (36, 37). However, in neoplastic tissues, the tight junctions between tumor cells are weak, decreasing the mucus barrier and increasing the epithelium permeability, thus facilitating the penetration of bacteria into the tissue (37). This may lead to a significantly large number of bacteria within the CRC tissue, and therefore higher risk of contamination of the PDO cultures. Indeed, here we showed that no washing step prior to CRC tissue processing results in high risk of microbial contamination in organoid cultures. In line with previous reports (10, 11), washing the tissue with PBS and P/S-containing solution reduces the risk of microbial contamination, but was not sufficient to completely prevent it.

Importantly, our data show that washing CRC tissues with a solution containing Primocin prevents microbial contamination in CRC-PDOs. We propose that laboratories involved in CRC PDO generation can benefit from our fast and easy-to-apply protocol. The three times 5-min wash of the CRC tissue in a cold solution containing PBS-Primocin (0.1 mg/ml), while reducing the time where tissue-derived cells are outside an appropriate culture condition (compare longer washing protocols), is sufficient to prevent microbial contamination.

Of note, we were able to keep the growing non-contaminated CRC-PDOs up to 10 passages without any new contamination event, indicating that the impact of our washing steps has a long-lasting effect.

Patients with neoplastic diseases are predisposed to invasive fungal infections, mainly related to *Candida aspergillus* species and other yeast-like fungi. Different factors may be responsible for this (1): prolonged granulocytopenia and disruption of mucosal and cutaneous barriers that results from intensive cytotoxic chemotherapy or ablative radiation therapy (2), impaired cell-mediated immunity that is caused by use of corticosteroids, and (3) the use of broad-spectrum antibiotics in the clinics prior to surgery, as they can alter the equilibrium among endogenous mucosal bacteria and facilitate the overgrowth of pathogenic species (38, 39). In our cohort of PDOs, only 1 out of 10 contaminated cases was due to fungal contamination. The tissue in this case was obtained from a patient (P135) diagnosed with rectal cancer who was treated with both radiotherapy and chemotherapy prior to surgery. Again, only in those samples washed with Primocin-containing solution was no fungal contamination observed.

We additionally found that a 5-min (3×) washing step with a P/S-containing solution is sufficient to reduce the percentage of living cells during tissue processing, therefore impairing the chances of generating viable PDOs (40). Given the fact that we did not test different P/S concentrations, we cannot rule out the possibility that reducing its concentration in the washing solution will increase cell viability. However, while lower concentration may likely reduce the cytotoxic effect, we cannot ensure the anti-contaminant activity.

It has been reported that treatment of stem cells with P/S for 24 h is sufficient to decrease cell viability by 30% (41). Moreover, it has been shown that the use of antibiotics impacts cell transcriptional activity (42) and differentiation (43). These alterations impact cellular hierarchy within the organoids and affect stem cell viability and growth rate, whose functionality is at the basis of organoid generation (40, 44–46). Indeed, ChIP-seq analysis of H3K27ac identified approximately 10,000 sites that were enriched near genes involved in cell differentiation, tRNA modification, nuclease activity, and protein dephosphorylation, in cells treated with P/S (42). Based on this evidence, we suggest that removing this reagent from both washing and culture media could bring benefit to cell and organoid cultures.

Different commensal Mycoplasma species have been isolated from human, for example, from oropharynx and vaginal mucosa and, although mycoplasma is not normally present within the intestinal flora, it has been linked to the onset and development of CRC (47–49). Interestingly, the study performed by Huang et al. (47) reported that 55% of patients with colon carcinoma had a mycoplasma infection and that, overall, gastric and colon cancers with high differentiation had a higher mycoplasma infection ratio than those with low differentiation. Moreover, other research pointed out that mycoplasma infection is linked to and responsible for prostate cancer and CRC (50), further supporting the presence of this bacteria in neoplastic tissues.

Our results show that mycoplasma was not present after PDO generation in any of the 5 conditions included in the present work, indicating that this may be due to the anti-mycoplasma activity of Primocin added in the culture medium and not in the washing solution.

We believe that our approach offers a balanced solution to avoid both the absence and the abuse of antibiotics within the washing solution. The exclusion of antibiotics increases the risk of incurring bacterial contamination (11, 14), while the use of a mixture of several antibiotics increases the chance of interfering with transcriptional activity (42) and cell differentiation (43), important for cell viability and the establishment of a cellular hierarchy in organoids (45).

In this study, we have shown that the addition of antibiotics to the growth medium is not enough to protect CRC PDOs from both bacterial and fungal-yeast contamination. Rather than focusing on further improving the protocol for PDO derivation, in this study, we aimed to standardize the procedure of the washing step and to promote the reduced use of antibiotics, especially P/S, as this has been demonstrated to have negative effects on stem cells’ functional states. We propose an easy-to-apply tissue washing protocol with a Primocin-containing solution as a step performed prior to CRC organoid generation in order to avoid microbial contamination.

## Data Availability Statement

The original contributions presented in the study are included in the article/**Supplementary Material**. Further inquiries can be directed to the corresponding authors.

## Ethics Statement

The studies involving human participants were reviewed and approved by Ethics Committee of Basel, EKBB, no. 2019-02118. The patients/participants provided their written informed consent to participate in this study.

## Author Contributions

SP and MC-L conceived and supervised the study. MM performed the experiment. SSt, SSo, OK, RD, AZ, MB, RP, and MF provided the samples and critically discussed the results. LF and ST-M provided sample information. GB, FP, and JG critically discussed the results. CE (experienced pathologist) performed the microbial identification. MM, CN, MC-L, and SP interpreted the results and wrote the manuscript. All authors contributed to the article and approved the submitted version.

## Funding

SP was supported by the Swiss Cancer League (KFS-4988-02-2020-R), by the Theron Foundation, Vaduz (LI), and by the Surgery Department of the University Hospital Basel. The funding bodies had no role in study design; in the collection, analysis, and interpretation of data; in the writing of the report; and in the decision to submit the article for publication.

## Conflict of Interest

Authors AZ and SR were employed by Viollier AG.

The remaining authors declare that the research was conducted in the absence of any commercial or financial relationships that could be construed as a potential conflict of interest.

## Publisher’s Note

All claims expressed in this article are solely those of the authors and do not necessarily represent those of their affiliated organizations, or those of the publisher, the editors and the reviewers. Any product that may be evaluated in this article, or claim that may be made by its manufacturer, is not guaranteed or endorsed by the publisher.

## Supplementary Material

Supplementary Figure 1 Mycoplasma analysis in PDOs culture. Representative results of PCR analysis performed on organoid growing medium. Each number represents one of the 5 washing conditions used in the present study: (1) no wash, (2) PBS, (3) P/S, (4) Primocin, (5) Primocin + P/S. Numbers in red indicate the washing conditions with positive for bacterial contamination. CTRL: control.
