# Supplementary figures and images for: Standardizing Patient-Derived Organoid Generation Workflow to Avoid Microbial Contamination From Colorectal Cancer Tissues
Source: Front Oncol. 2022 Jan 10;11:781833. doi: 10.3389/fonc.2021.781833 (PMC8784867; doi:10.3389/fonc.2021.781833)

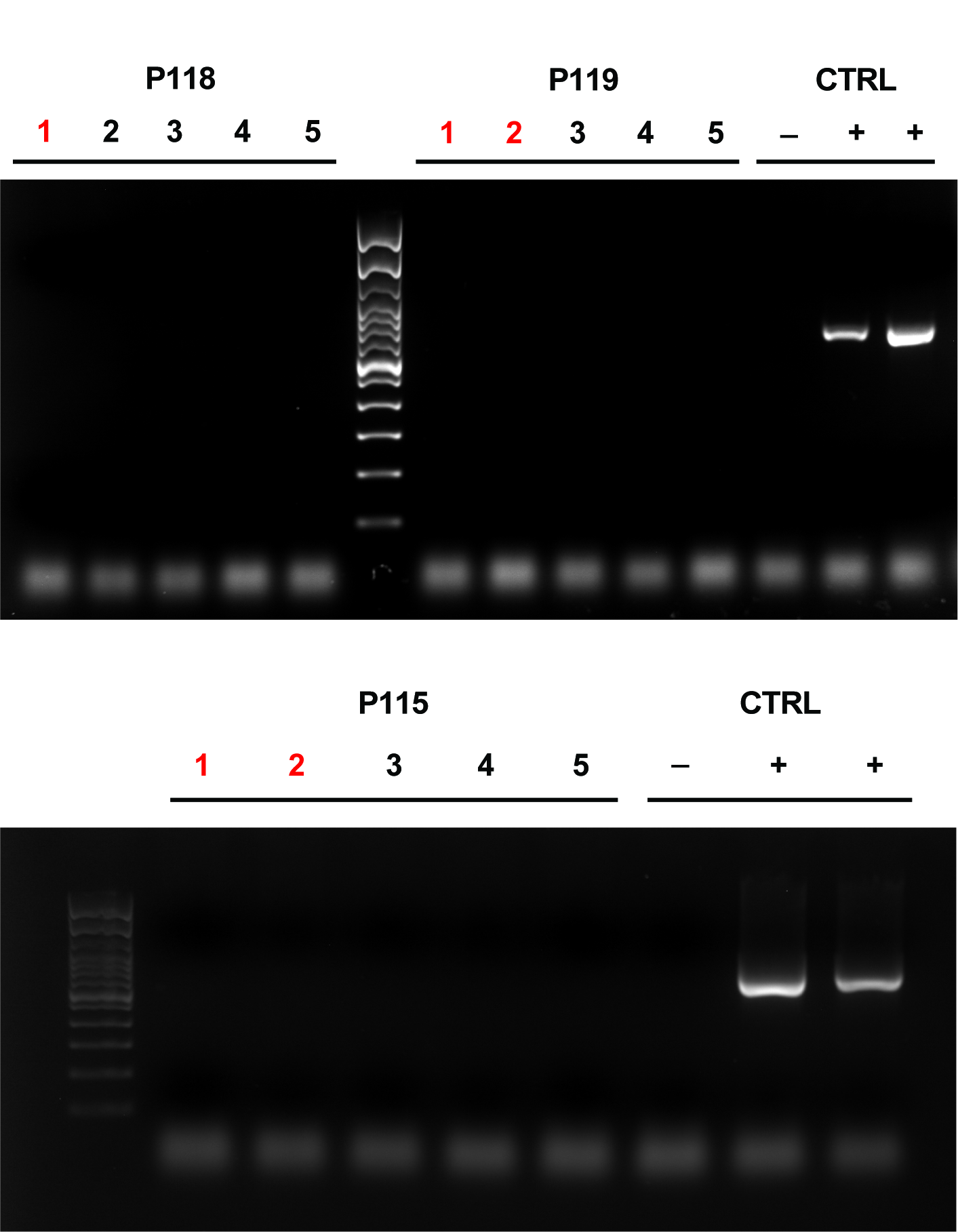

Supplement: Supplementary Figure 1 — Mycoplasma analysis in PDOs culture. Representative results of PCR analysis performed on organoid growing medium. Each number represents one of the 5 washing conditions used in the present study: (1) no wash, (2) PBS, (3) P/S, (4) Primocin, (5) Primocin + P/S. Numbers in red indicate the washing conditions with positive for bacterial contamination. CTRL: control. [file Image_1.tif]
